# *Actinomyces funkei* bacteraemia and infected pulmonary cavities in an intravenous drug user: a case report

**DOI:** 10.1186/s41182-024-00610-7

**Published:** 2024-08-26

**Authors:** Tanaraj Perinpanathan, Katherine Beckett, Chris Smith

**Affiliations:** 1 London North West University Healthcare NHS Trust, London, UK; 2 Imperial College Healthcare NHS Trust, London, UK; 3 Clinical Research, Nagasaki University, Nagasaki, Japan; 4 School of Tropical Medicine and Global Health, Nagasaki University, Nagasaki, Japan; 5 Department of Clinical Research, London School of Hygiene and Tropical Medicine, London, UK

**Keywords:** *Actinomyces funkei*, Intravenous drug user, Septic emboli, Matrix-assisted laser desorption/ionization time-of-flight

## Abstract

**Background:**

*Actinomyces* spp. are most commonly found in human commensal flora; however, they have also been shown to cause suppurative infections. We present a case of a rare *Actinomyces funkei* bacteraemia from an infected deep vein thrombosis in a patient who went on to develop pulmonary cavities secondary to septic emboli. Infected thrombi and septic emboli have been associated with other *Actinomyces* spp. in the literature, often posing a diagnostic challenge and, in some cases, causing drastic clinical deterioration in patients. The literature regarding *Actinomyces funkei* is scarce and to our knowledge there are no reports of a relationship between this *Actinomyces* subspecies and infected thrombi or septic emboli.

**Case presentation:**

The patient was a 39-year-old known intravenous drug user who presented with a groin injecting site sinus and systemic symptoms. The bacteria was first observed by gram staining of a blood culture sample after 48 h of incubation and the species was identified using matrix-assisted laser desorption ionization time-of-flight (MALDI-TOF) as *Actinomyces funkei.* Sputum cytology/histology with cell block revealed a branching gram-positive species suspicious of slow growing bacteria or fungus. CT imaging of his lower limb and chest revealed an extensive DVT with inflammatory changes and pulmonary cavities respectively. The patient was treated with Ceftriaxone before being discharged with a 6-month course of Linezolid. He made a good recovery with reduction in size of the cavitating lung lesions on follow-up imaging.

**Conclusions:**

This case report presents a difficult-to-diagnose bacterial infection in an intravenous drug user, complicated by bacteraemia and secondary septic emboli. Relatively little is known about *Actinomyces funkei*, and therefore this report aims to increase clinician awareness of diagnosis, management, and complications.

## Background

*Actinomyces* spp. are most commonly found in the human commensal flora of the oropharynx, gastrointestinal tract, and urogenital tract. More than 40 different species have been identified, usually causing suppurative infections at various anatomical sites, often forming cold abscesses [1].

*Actinomyces funkei* was first described in 2001, following isolation from a female intravenous drug user (IVDU) with a history of endocarditis [2]. It has since been reported in the literature in eight other human cases, isolated from abscesses, wound swabs, biopsy, and blood cultures [2, 4, 5]. To our knowledge, there are no published reports documenting a relationship between *Actinomyces funkei* and infected thrombi or septic emboli. It is noted that both have been associated with other *Actinomyces* spp. in the literature, often posing a diagnostic challenge and, in some cases, causing drastic clinical deterioration to the patient [6, 7].

Here, we present a case of a 39-year-old male with two sequential presentations; initially with an infected DVT followed by pulmonary infected cavities, with microbiology confirming *Actinomyces funkei* as the causative pathogen.

## Case presentation

The patient was a 39-year-old male intravenous drug user, who presented twice to a UK hospital with fevers and associated symptoms. He had a 10-year intermittent history of injecting heroin, with his last use three weeks prior to his first admission. He reported re-using needles but never sharing them with others. His past medical history included two previous admissions for cellulitis several years ago. He had a 20-pack-year smoking history and had recently stopped drinking alcohol after previous excess consumption. He had no history of recent travel. He was last screened for HIV, Hepatitis B and C a year ago, all of which were negative.

On first admission, he was febrile, with left leg swelling and associated brown discharge at a groin injection site. An infected deep vein thrombosis (DVT) was visualised in the external iliac vein with florid inflammatory changes on CT Angiogram. Blood cultures were negative. Chest X-ray was normal. He showed clinical improvement on intravenous flucloxacillin and anticoagulation and was discharged on oral flucloxacillin and apixaban with follow up in two weeks.

The patient returned to hospital 12 days later with a three day history of a reemergent fever, rigors and sweating. He reported shortness of breath on exertion with reduced exercise tolerance and pleuritic chest pain. He described a new cough, productive of brown offensive sputum, but no haemoptysis.

The following information relates to the patient's second admission.

### Clinical examination

The patient had a temperature of 38.8 °C, heart rate of 96 bpm, blood pressure of 130/75 mmHg, respiratory rate of 26 breaths per minute and oxygen saturation of 92% on room air. He was alert and oriented. He was clinically euvolaemic with no peripheral stigmata of endocarditis. No murmurs were audible on auscultation; however, coarse inspiratory and expiratory crepitations were heard in the right upper zone of the chest. No abdominal or neurological signs were noted and there was no spinal tenderness. Further examination of the groin injection site revealed persistent purulent discharge. Dental examination showed poor dentition, but without obvious evidence of dental infection.

### Investigations

#### Blood tests

Key blood results are noted in Table 1. The patient was mildly anaemic, with elevated inflammatory markers and a mild eosinophilia. He had a stage 1 acute kidney injury (AKI). His HIV p24 and antibody test was negative while hepatitis B serology showed evidence of previous vaccination. All other blood results were within normal range.

**Table 1:** Blood test results

Blood test	Result	Normal range	Blood test	Result	Normal range
Haemoglobin (g/L)	114	130–180	Na (mmol/L)	137	135–145
White Cell Count (× 10^9^/L)	13.6	4–11	K (mmol/L)	4.2	3.5–5.0
Neutrophils (× 10^9^/L)	8.6	2–7.5	Urea (mmol/L)	8.0	2.5–7.8
Lymphocytes (× 10^9^/L)	3.7	1.5–4.0	Creatinine (μmol/L)	130	59–104
Eosinophils (× 10^9^/L)	0.45	0.0–0.4	ALP (U/L)	128	30–130
Platelets (× 10^9^/L)	427	150–450	ALT (U/L)	12	< 31
Haematocrit (Hct)	0.48	0.4–0.52	Bilirubin (μmol/L)	6	< 21
CRP (mg/L)	286	< 6	HIV Antibody and p24	Negative	–
Lactate (mmol/L)	1.2	0.5–2.2	Hep B HBsAg	Negative	–
Prothrombin time (s)	13.7	10–14	Hep B Anti-HBs	Positive	–
APTT (s)	35.2	24–37	Hep B HBcAg	Negative	–

#### Imaging

**Fig. 1:**
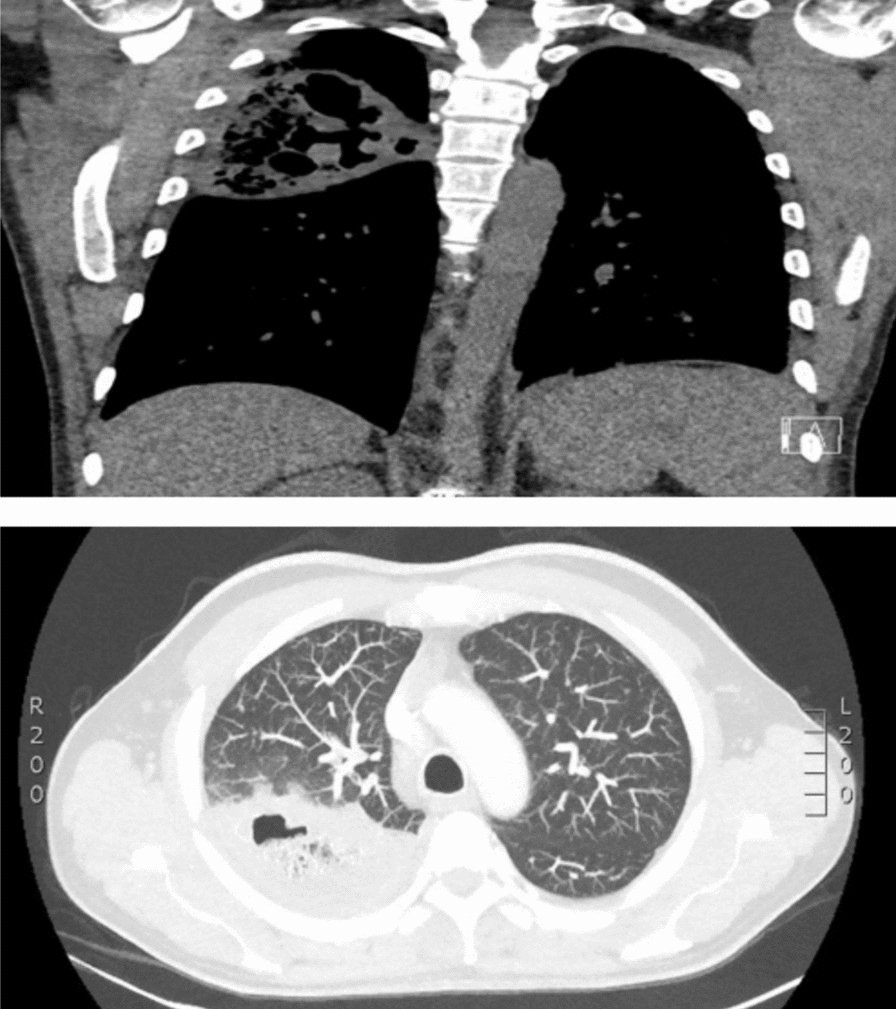
CT thorax showing large thick-walled gas-filled cavitating lesions in the posterior aspect of the right upper lobe measuring approximately 5.3 × 7.7 × 9.4 cm. Multiple smaller cavitating lesions were seen in the left upper and lower lobes, with patchy regions of ground-glass change

Chest X-ray showed large consolidation in the right upper lobe and left retrocardiac opacification. CT Thorax revealed large thick-walled gas-filled cavitating lesions in the posterior aspect of the right upper lobe measuring approximately 5.3 × 7.7 × 9.4 cm (Fig. 1). Multiple smaller cavitating lesions were seen in the left upper and lower lobes, with patchy regions of ground-glass change.

Repeat CT Lower Limb Angiogram showed extension of the inflammation more distally from the left external iliac vessel to include the superficial femoral vein, demonstrated by vessel wall thickening, adjacent inflammatory stranding and intraluminal gas locules.

Echocardiography showed normal valvular morphology with no vegetations. The patient had normal sized ventricles with good associated function and no pericardial effusion.

#### Microbiology

Biofire® respiratory nasopharyngeal PCR panel and three AFB sputum samples all returned negative. MCS swabs from the purulent groin sinus showed mixed growth of indeterminate significance. Sputum cytology/histology with cell block revealed a branching gram-positive species suspicious of slow-growing bacteria or fungus.

Initial blood cultures were negative. However, growth of a gram-positive bacteria was noted in one of the cultures after 48 h, later identified by matrix-assisted laser desorption ionization time-of-flight (MALDI-TOF) as *Actinomyces funkei*.

### Management approach

The patient was transferred to a medical high-dependency unit. He was initially treated with intravenous fluids and broad-spectrum antibiotics (piperacillin–tazobactam IV 4.5 g, 8-hourly), remaining on these due to the diagnostic uncertainty. Once *Actinomyces funkei* was identified, his antibiotics were changed to Ceftriaxone IV 2 g, 12-hourly. He was discharged on a prolonged course of antimicrobial therapy (Linezolid 600 mg twice daily for six months).

He remained on treatment dose anticoagulation for three months. No further sites of seeding from haematological spread were noted on subsequent imaging. He was referred to the community drug and alcohol team on discharge.

The patient was followed up in the outpatient clinic and serial chest X-rays showed reduction in the size of the cavitating lung lesions. He remained asymptomatic. He had returned to work and continued to abstain from further intravenous drug use with the help of community support.

## Discussion

*Actinomyces* spp. are non-motile, filamentous, anaerobic Gram-positive bacilli [3]. A literature review of *Actinomyces* spp. infections in IVDU identified only eight case reports, with zero cases of septic emboli secondary to DVT [2, 4, 5]. Of the eight case studies, only one patient was an intravenous drug user. This case report, in which the patient developed infective endocarditis, was the first to describe the subspecies [2]. Two subsequent reports describe isolation of the bacterium from soft-tissue abscesses, surgical site infections and pressure sores [4, 5]. In addition, *Actinomyces funkei* was isolated from a liver abscess in one patient, with a suspected intestinal focus of infection following diverticular surgery [4]. We believe that this case report is the first to describe an *Actinomyces funkei* infection from an infected deep vein thrombosis and the first to report on the formation of cavitation lung lesions secondary to septic emboli (see Appendix 1 for literature review search terms).

There is difficulty in isolating *Actinomyces* spp. as it is a fastidious, slow-growing organism that may only be detected after 5–10 days of anaerobic inoculation [3]. Several case reports describe a difficulty in identifying the species, with upwards of 50% of cases being culture negative [8]. There are a several possible causes for this; previous antibiotic use, short incubation periods or the commonly polymicrobial nature of infections whereby other bacteria may inhibit growth [3, 8]. A Gram stain of the specimen may be more sensitive than culture especially if the patient has received antibiotics, as in our case. The advent of MALDI-TOF has enabled easier diagnosis of rarer pathogens. MALDI-TOF is based on the sample mass spectrometry data [9], a characteristic spectrum of peptides is generated for each analyte, and then matched against a database of spectrometry profiles [10].

*Actinomyces* spp. are a part of the genital flora which may account for why injecting into a groin site may have introduced the pathogen into the bloodstream of this patient. Furthermore, in five of the eight case reports of *Actinomyces funkei* infection in the literature, the bacteria was isolated from the groin region [4]. While *Actinomyces* spp. from skin can contaminate samples and mislead diagnosis, this is unlikely in this case as the species was identified through two different means, gram stain and MALDI-TOF, and by two different mediums, blood and sputum.

Once diagnosis is made, management is relatively straightforward. Most *Actinomyces* spp. are susceptible to penicillin [3, 8]. However, there can be discrepancies between in vitro and in vivo susceptibility, especially given the protection of abscess wall formation and, in this case, recent treatment with flucloxacillin. Thus, a decision was made to treat the patient with a third-generation cephalosporin with good respiratory tract penetration. The recommended duration of treatment is not clear; most case studies report 6–12 months of antibiotic therapy, although successful outcomes have been reported in early infections with 3-month courses [10–12].

## Conclusion

This case report presents a difficult-to-diagnose bacterial infection caused by *Actinomyces funkei* in an intravenous drug user, resulting in bacteraemia and secondary septic emboli. Relatively little is known about *Actinomyces funkei*, and therefore this report aims to increase clinician awareness of diagnosis, management, and complications.

## Data Availability

Not applicable.

## Appendix 1

**Table:**

Search number	Query	Search details	Results
5	#2 AND #4	("schaalia funkei"[Supplementary Concept] OR "schaalia funkei"[All Fields] OR "actinomyces funkei"[All Fields]) AND ("drug users"[MeSH Terms] OR ("drug"[All Fields] AND "users"[All Fields]) OR "drug users"[All Fields] OR "ivdu"[All Fields] OR ("drug users"[MeSH Terms] OR ("drug"[All Fields] AND "users"[All Fields]) OR "drug users"[All Fields] OR ("intravenous"[All Fields] AND "drug"[All Fields] AND "user"[All Fields]) OR "intravenous drug user"[All Fields]) OR "PWID"[All Fields] OR ("drug users"[MeSH Terms] OR ("drug"[All Fields] AND "users"[All Fields]) OR "drug users"[All Fields] OR ("people"[All Fields] AND "who"[All Fields] AND "inject"[All Fields] AND "drugs"[All Fields]) OR "people who inject drugs"[All Fields]))	0
4	Actinomyces funkei	"schaalia funkei"[Supplementary Concept] OR "schaalia funkei"[All Fields] OR "actinomyces funkei"[All Fields]	6
3	#1 AND #2	("drug users"[MeSH Terms] OR ("drug"[All Fields] AND "users"[All Fields]) OR "drug users"[All Fields] OR "ivdu"[All Fields] OR ("drug users"[MeSH Terms] OR ("drug"[All Fields] AND "users"[All Fields]) OR "drug users"[All Fields] OR ("intravenous"[All Fields] AND "drug"[All Fields] AND "user"[All Fields]) OR "intravenous drug user"[All Fields]) OR "PWID"[All Fields] OR ("drug users"[MeSH Terms] OR ("drug"[All Fields] AND "users"[All Fields]) OR "drug users"[All Fields] OR ("people"[All Fields] AND "who"[All Fields] AND "inject"[All Fields] AND "drugs"[All Fields]) OR "people who inject drugs"[All Fields])) AND ("actinomyce"[All Fields] OR "actinomyces"[MeSH Terms] OR "actinomyces"[All Fields])	19
2	(IVDU) OR (Intravenous drug user) OR (PWID) OR (People who inject drugs)	"drug users"[MeSH Terms] OR ("drug"[All Fields] AND "users"[All Fields]) OR "drug users"[All Fields] OR "ivdu"[All Fields] OR ("drug users"[MeSH Terms] OR ("drug"[All Fields] AND "users"[All Fields]) OR "drug users"[All Fields] OR ("intravenous"[All Fields] AND "drug"[All Fields] AND "user"[All Fields]) OR "intravenous drug user"[All Fields]) OR "PWID"[All Fields] OR ("drug users"[MeSH Terms] OR ("drug"[All Fields] AND "users"[All Fields]) OR "drug users"[All Fields] OR ("people"[All Fields] AND "who"[All Fields] AND "inject"[All Fields] AND "drugs"[All Fields]) OR "people who inject drugs"[All Fields])	60,895
1	Actinomyces	"actinomyce"[All Fields] OR "actinomyces"[MeSH Terms] OR "actinomyces"[All Fields]	8,824

## Abbreviations

IVDU: Intravenous drug user
DVT: Deep vein thrombosis
AKI: Acute kidney injury
MALDI-TOF: Matrix-assisted laser desorption/ionization time-of-flight
